# Efficient polygalacturonase production from agricultural and agro-industrial residues by solid-state culture of *Aspergillus sojae* under optimized conditions

**DOI:** 10.1186/2193-1801-3-742

**Published:** 2014-12-16

**Authors:** Doreen Heerd, Sonja Diercks-Horn, Marcelo Fernández-Lahore

**Affiliations:** Downstream Processing Laboratory, Jacobs University Bremen gGmbH, Campus Ring 1, 28759 Bremen, Germany

**Keywords:** Polygalacturonase, *Aspergillus sojae*, Design of Experiment, Solid-state fermentation, Agro-industrial residues

## Abstract

Previously identified fungal pectinase producers of the species *Aspergillus sojae* were used for optimization of polygalacturonase production in solid-state fermentation applying Design of Experiment. The effects of media composition and several process parameters, like inoculum size, moisture level, incubation time and temperature on polygalacturonase activity were studied in screening and optimization investigations. Utilization of agricultural and agro-industrial by-products provided the establishment of a cost-efficient and sustainable process for enzyme production. Comparison of pectinase production by *A. sojae* ATCC 20235 and *A. sojae* CBS 100928 under optimized conditions yielded 6.9 times higher polygalacturonase activity by *A. sojae* ATCC 20235. Highest enzyme yield (909.5 ± 2.7 U/g) was obtained by *A. sojae* ATCC 20235 after 8 days at 30°C applying 30% sugar beet pulp as inducer substrate in combination with wheat bran as medium wetted at 160% with 0.2 M HCl. Furthermore, an overview of pectinolytic enzyme activities present in the extracts of both strains is provided. Protein profiles of both strains are given by SDS-PAGE electrophoresis, as well as zymograms for pectinolytic enzymes in comparison to commercial pectinase preparations.

**Electronic supplementary material:**

The online version of this article (doi:10.1186/2193-1801-3-742) contains supplementary material, which is available to authorized users.

## Background

The biotechnological potential of pectinolytic enzymes is well known due to their various industrial applications wherever degradation of pectic substances is required. This includes food related processes like fruit juice clarification, tissue maceration, wine clarification, coffee and tea fermentation and many others (Kashyap et al. 2001). It has been reported that pectinases have a share of almost 5% of global enzyme sales (Alimardani-Theuil et al. 2011). Commercial pectinases used in food industry normally contain a mixture of enzymes that split pectic compounds; which traditionally includes PG (polygalacturonase), PL (pectin lyase) and PME (pectin methylesterase) (Del Cañizo et al. 1994). Pectinolytic enzyme production occupies about 10% of the worldwide manufacturing of enzyme preparations (Semenova et al. 2006). Industrial production of microbial pectinolytic enzymes is mainly done by filamentous fungi, especially *Aspergillus niger* (Naidu and Panda 1998). *Aspergillus* species produce a large number of enzymes particularly involved in the degradation of pectic substances (van den Brink and de Vries 2011). However, the variety of enzyme sets differs between fungal species (Benoit et al. 2012).

The *koji* molds, *Aspergillus oryzae* and *Aspergillus sojae*, are often associated with a long history of safe use in traditional food fermentations and several *Aspergillus*-derived food additive products have already obtained a GRAS (generally recognized as safe) status from regulatory authorities (Heerikhuisen et al. 2005, Jørgensen 2007), which supports the potential of these strains as production organisms for pectinolytic enzymes used in food industry.

Filamentous fungi are known to produce pectinolytic enzymes in submerged fermentation (SmF), as well as in solid-state fermentation (SSF) processes. They are capable of synthesizing and secreting large quantities of certain proteins into the extracellular medium. Degradation and utilization of diverse biopolymers such as starch, cellulose or pectin enables cultivation of *Aspergillus* species on agricultural and agro-industrial residues, which can be used as low-cost substrates for microbial enzyme production in SSF processes (Nigam and Pandey 2009). The application of agricultural and agro-industrial by-products, such as apple pomace or sugar beet pulp, offers a wide range of alternative substrates and helps to solve disposal problems of these by-products. The apple pomace is mainly composed of insoluble carbohydrates such as cellulose, hemicelluloses and lignin. Traditionally, apple pomace and citrus peels are used as raw materials for pectin production. Citrus peels consist of approximately 24% of pectic substances on α-D-galacturonic acid basis (Yapo et al. 2007). Alternatively, high content in pectins (20-25%), its availability and low-cost make sugar beet pulp a potential source of pectins. Sugar beet pulp is mainly composed of (% on dry basis) pectin, 28.7; cellulose, 20; hemicellulose, 17.5; protein, 9.0; and lignin 4.4 (Jacob 2009). Since the pectin content of beet pulp or apple pomace is high it can be used for the microbial production of pectinolytic enzymes without adding pectinaceous material as inducer. However, the most potent solid agro-industrial by-product for biotechnological pectinolytic enzyme production, used in combination or without pectinase inducers, is wheat bran, which is composed predominantly of non-starch carbohydrates, starch and crude proteins (Jacob 2009).

Utilization of low-cost agro-industrial residues offers potential benefits for SSF, which is attractive for implementation of sustainable bioprocesses. Further advantages of SSF processes are lower energy requirement associated with higher product yields and less wastewater production with lesser risk of bacterial contamination. Nevertheless, there are many factors which have a critical influence on the process development in SSF, such as selection of microorganism and substrate or optimum physical-chemical and biological process parameters (Thomas et al. 2013). In view of this, optimization of the SSF process for PG production by *A. sojae* was targeted applying statistical design tools.

A previous study demonstrated already the potential of *A. sojae* for pectinase production in SSF (Heerd et al. 2012). A further study about application of strain improvement methods revealed the increase of pectinase production by *A. sojae* applying a classical mutation and selection strategy (Heerd et al. 2014). The present study describes the SSF process optimization for enhancing the pectic acid-degrading activity, which is referred to as PG activity, using statistical design techniques. Response surface methodology (RSM) was applied for optimization of SSF utilizing agricultural and agro-industrial by-products as commercial substrates for the establishment of a sustainable bioprocess. Carbohydrate active enzymes of the pectinase enzyme-complex were investigated comparing the production by two fungal strains of *A. sojae* under optimized conditions and characterizing the pectinolytic enzyme sets on the basis of their substrate degrading mode.

## Materials and methods

### Materials

Dried bitter orange peel (*Cortex Aurantii Fructus amar. conc.*) was purchased from Heinrich Klenk GmbH & Co. KG (Schwebheim, Germany) and was ground to small particles (of varied size) in a coffee mill. Nordzucker AG (Uelzen, Germany) provided two different kinds of sugar beet pulp pellets: molassed (with approximately 30% molasses) and unmolassed. Pelletized pulp (dry matter > 89%) was ground to small particles due to easier handling in SSF on laboratory scale. Apple pomace, a by-product from processing apples, was a heterogeneous mixture of different kinds of apples, like Elstar, Jonagold, Jonagored or Braeburn and was obtained from Döhler GmbH (Neuenkirchen, Germany). It had a moisture content of approximately 73% (moisture content on wet-basis), determined by drying at 105°C until constant weight. All by-products in this study were used in combination with wheat bran, which was obtained as fine bran (90% < 630 μm) from Bremer Rolandmühle Erling GmbH & Co. KG (Bremen, Germany).

All chemicals were purchased from AppliChem GmbH (Darmstadt, Germany), except dithiothreitol (DTT) was purchased from Carl Roth GmbH & CO. KG (Karlsruhe, Germany). Substrates for detection of pectinolytic activities, e.g. pectin, polygalacturonic acid and polygalacturonic acid sodium salt, as well as the chemical sodium arsenate dibasic heptahydrate, and pectinase from *A. niger* were purchased from Sigma-Aldrich Chemie GmbH (Steinheim, Germany). Fructozym P was obtained from ERBSLÖH Geisheim AG (Geisheim, Germany).

### Microorganisms

*A. sojae* CBS 100928 was obtained from the Centraalbureau voor Schimmelcultures (CBS) (Utrecht, Netherlands) and *A. sojae* ATCC 20235 was provided by **İ** zmir Institute of Technology, **İ** zmir, Turkey. Propagation was done on agar plates according to the specifications given in Heerd et al. (2012). It has to be noted that *A. sojae* ATCC 20235, which is still deposited as *A. sojae* at the American Type of Culture Collection (ATCC), did not meet the requirements to be classified as *A. sojae* on the basis of morphological parameters (Ushijima et al. 1982), and has been reclassified as *A. oryzae* based on the *alpA* restriction fragment length polymorphism (RFLP) (Heerikhuisen et al. 2005).

### Inoculum

The spore suspensions used as inoculum were obtained from molasses agar slants containing: glycerol (45 g/L), molasses (45 g/L), peptone (18 g/L), NaCl (5 g/L), KCl (0.5 g/L), FeSO_4_ · 7H_2_O (15 mg/L), KH_2_PO_4_ (60 mg/L), MgSO_4_ (50 mg/L), CuSO_4_ · 5H_2_O (12 mg/L), MnSO_4_ · H_2_O (15 mg/L) and agar (20 g/L). Slants were incubated at 30°C for 1 week. Spores were harvested from the slants using sterile Tween 80 water (0.02%) and counted in a Thoma counting chamber to adjust the spore concentrations according to the experimental design (see section Experimental Design).

### Culture medium and growth conditions

SSF was performed in 300-mL culture flasks containing 10 g solid media wetted with diluted HCl at the respective concentrations according to the experimental design. The described moisture levels in all experimental set-ups were calculated as dry basis moisture content as described in Heerd et al. (2014). Culture flasks containing the wetted media were sterilized at 121°C for 20 min. Flasks were inoculated with 1 mL of spore suspension containing the desired amount of total spores and incubated according to the experimental design (see section Experimental Design).

### Experimental design

Experiments were designed and evaluated utilizing the MODDE 9.0 software package, supplied by Umetrics AB, Umeå, Sweden. Exo-PG activity was the response value in all experimental setups.

#### PG production by A. sojae ATCC 20235

In the screening part of this study the effects of temperature (X_T_), inoculum size (X_Is_), time (X_t_), HCl concentration (X_HCl_) and inducer substrate (X_S_) on PG production were investigated using a complemented D-optimal design with two replicated center-points of each inducer substrate, giving a total number of 24 trials (Table 1). An estimate of the main effect was obtained by evaluating the difference in process performance caused by a change from the low to the high levels of the corresponding variable. The moisture content was fixed at 120%.

**Table 1:** **D-optimal design and experimental results of exo-PG activity in the first screening step of** ***A. sojae*** **ATCC 20235**

	Experimental factors					Response
	Inducer substrate	Temperature (°C)	Inoculum (Total spores)	Time (d)	HCl concentration (mM)	exo-PG activity (U/g)
1	Orange peel	32	3 × 10^7^	3	50	30.3
2	Orange peel	32	3 × 10^7^	5	50	21.5
3	Orange peel	24	10^4^	3	300	-
4	Orange peel	24	10^4^	5	300	-
5	Apple pomace	24	10^4^	3	50	20.0
6	Apple pomace	32	10^4^	3	50	20.1
7	Apple pomace	24	3 × 10^7^	5	300	216.3
8	Apple pomace	32	3 × 10^7^	5	300	223.8
9	Sugar beet pulp	24	3 × 10^7^	3	50	58.5
10	Sugar beet pulp	32	10^4^	5	50	94.9
11	Sugar beet pulp	24	3 × 10^7^	3	300	-
12	Sugar beet pulp	32	10^4^	5	300	-
13	Sugar beet with molasses	24	10^4^	5	50	76.7
14	Sugar beet with molasses	24	3 × 10^7^	5	50	30.5
15	Sugar beet with molasses	32	10^4^	3	300	-
16	Sugar beet with molasses	32	3 × 10^7^	3	300	-
17 – 18*	Orange peel	28	1,5 × 10^7^	4	175	140.3 ± 3.8
19 – 20*	Apple pomace	28	1,5 × 10^7^	4	175	243.0 ± 10.3
21 – 22*	Sugar beet pulp	28	1,5 × 10^7^	4	175	407.5 ± 13.9
23 – 24*	Sugar beet with molasses	28	1,5 × 10^7^	4	175	291.2 ± 1.7

*The standard variation between the values of each center point repetition was below 5%.

A 2^4^ full factorial design with two replicated center-points for each of the two selected inducer substrates (sugar beet pulp and apple pomace), giving a total of 20 trials, was used to further explore the effects of the two inducer substrates and three more variables (inducer concentration, HCl concentration and time) (Table 2). Experiments were conducted at 28°C applying a moisture level of 120% and an inoculum size of 2 × 10^7^ spores per trial.

**Table 2:** **Full factorial design and experimental results of exo-PG activity in the second screening step of** ***A. sojae*** **ATCC 20235**

Trial	Experimental factors				Response
	Inducer substrate	Inducer concentration (%)	HCl concentration (mM)	Time (d)	exo-PG activity (U/g)
1	Apple pomace	10	0.1	3.5	28.8
2	Sugar beet pulp	10	0.1	3.5	68.2
3	Apple pomace	40	0.1	3.5	57.5
4	Sugar beet pulp	40	0.1	3.5	104.2
5	Apple pomace	10	0.22	3.5	209.5
6	Sugar beet pulp	10	0.22	3.5	300.4
7	Apple pomace	40	0.22	3.5	165.5
8	Sugar beet pulp	40	0.22	3.5	-
9	Apple pomace	10	0.1	5.5	4.3
10	Sugar beet pulp	10	0.1	5.5	12.5
11	Apple pomace	40	0.1	5.5	38.1
12	Sugar beet pulp	40	0.1	5.5	88.8
13	Apple pomace	10	0.22	5.5	91.7
14	Sugar beet pulp	10	0.22	5.5	374.5
15	Apple pomace	40	0.22	5.5	225.2
16	Sugar beet pulp	40	0.22	5.5	-
17 – 18*	Apple pomace	25	0.16	4.5	118.2 ± 5.4
19 – 20*	Sugar beet pulp	25	0.16	4.5	277.5 ± 1.7

*The standard variation between the center point repetitions was below 5%.

In the optimization part, the settings of the two variables inducer concentration (X_S_) and moisture level (X_M_) were optimized using an experimental design, which was completed by manual addition of 7 trials to a D-optimal design for identification of the optimal region, giving a total of 18 trials (Table 3). Experiments were conducted at 28°C for 5 days with a total amount of 2 × 10^7^ spores per flask.

**Table 3:** **D-optimal design and experimental results of exo-PG activity in the medium optimization for** ***A. sojae*** **ATCC 20235**

Trial	Experimental factors		Response
	Inducer concentration (%)	Moisture level (%)	exo-PG activity (U/g)
1	10	85	124.1
2	35	85	270.1
3	10	135	371.2
4	35	135	552.3
5	10	110	252.4
6	35	110	413.5
7	22.5	85	221.9
8	22.5	135	493.6
9*	22.5	110	347.5
10*	22.5	110	328.5
11*	22.5	110	325.4
12	65	165	0
13	65	145	0
14	45	165	512.4
15	45	145	418.7
16	55	155	39.5
17	65	135	0
18	35	165	557.2

*The standard variation between the center point repetitions was below 3%.

Applying the optimized medium composition of 30% inducer substrate and a moisture level of 160%, the process parameters time (X_t_) and temperature (X_T_) were optimized using D-optimal design due to the manual addition of 3 experimental runs, giving a total of 14 trials, which were inoculated with 2 × 10^7^ spores per trial (Table 4).

**Table 4:** **D-optimal design and experimental results of exo-PG activity in the process optimization for** ***A. sojae*** **ATCC 20235**

Trial	Experimental factors		Response
	Time (d)	Temperature (°C)	exo-PG activity (U/g)
1	5	26	496.0
2	7	26	603.1
3	5	34	306.7
4	7	34	532.4
5	5	30	586.2
6	7	30	678.2
7	6	26	614.0
8	6	34	390.5
9*	6	30	619.4
10*	6	30	705.3
11*	6	30	680.3
12	8	26	693.9
13	8	30	847.3
14	8	34	651.0

*The standard variation between the center point repetitions was below 10%.

#### PG production by A. sojae CBS 100928

In the screening part, the effects of moisture level (X_M_), time (X_t_) and temperature (X_T_) on PG production by *A. sojae* CBS 100928 were studied using a full factorial design. The central points were performed in duplicate at the low and high level of the variable temperature due to the narrow temperature range, giving a total of 12 trials (Table 5).

**Table 5:** **Full factorial design and experimental results of exo-PG activity in the screening step of** ***A. sojae*** **CBS 10928**

Trial	Experimental factors			Response
	Moisture level (%)	Temperature (°C)	Time (d)	exo-PG activity (U/g)
1	80	24	4	23.6
2	160	24	4	36.4
3	80	30	4	31.6
4	160	30	4	39.6
5	80	24	8	33.9
6	160	24	8	106.0
7	80	30	8	34.1
8	160	30	8	104.2
9 – 10*	120	24	6	69.8 ± 1.8
11 – 12*	120	30	6	108.1 ± 5.9

*The standard variation between the center point repetitions was below 6%.

For optimization of the settings of two selected variables from the factorial design, moisture level (X_M_) and time (X_t_), a central composite face-centered (CCF) design with three replicates at the central point was applied, giving a total of 11 trials (Table 6).

**Table 6:** **Central composite face-centered design and experimental results of exo-PG activity in the optimization step of** ***A. sojae*** **CBS 100928**

Trial	Experimental factors		Response
	Moisture level (%)	Time (d)	exo-PG activity (U/g)
1	130	6	87.5
2	170	6	49.7
3	130	8	95.5
4	170	8	95.7
5	130	7	94.7
6	170	7	63.2
7	150	6	94.0
8	150	8	120.7
9*	150	7	93.8
10*	150	7	103.4
11*	150	7	102.0

*The standard variation between the center point repetitions was below 5%.

Following the findings for culturing conditions of *A. sojae* ATCC 20235, all experimental runs were inoculated with 2 × 10^7^ spores of *A. sojae* CBS 100928 per trial.

### Enzyme leaching and protein estimation

At the end of cultivation, the enzyme recovery was obtained by adding 50 mL distilled water into each flask and mixing in an incubator shaker (Innova 4230, New Brunswick Scientific) at 350 rpm, 24°C, for 60 min. The extract was separated from the fermented substrate by centrifugation at 4°C, 3220 × g, for 30 min. Enzyme activities and total protein concentration were determined in the supernatant.

Total extracellular protein content was measured according to the modified Bradford’s method (Bradford 1976), using the Coomassie Plus™ Protein Assay Kit (Pierce, Fischer scientific, Schwerte, Germany). The assay was performed in a microplate by detecting the absorbance at 595 nm using bovine serum albumin (BSA) as a standard. Soluble protein content was expressed as milligram per gram dry substrate (mg/g).

### Exo-pectinolytic activity measurement

#### Polygalacturonase assay

Exo-PG activity was assayed according to the procedure provided by Panda et al. (1999) with slight modifications as described in Heerd et al. (2014). One unit of exo-PG activity was defined as the amount of enzyme that catalyzes the release of 1 μmol of galacturonic acid per unit volume of supernatant per minute under the applied assay conditions. Exo-PG activity was expressed as unit per gram dry substrate (U/g).

#### Polymethylgalacturonase Assay

Exo-PMG activity was determined according to the method provided by Blandino et al. (2002) with slight modifications, as previously described in Heerd et al. (2012). One unit of exo-PMG activity was defined as the amount of enzyme that catalyses the release of 1 μmol of galacturonic acid per unit volume of supernatant per minute under the applied assay conditions. Exo-PMG activity was expressed as unit per gram dry substrate (U/g).

### Endo-pectinolytic activity measurement

Endo-enzyme activities were determined by measuring the decrease in viscosity of a substrate solution, either 2% (w/v) pectin for endo-PMG or 3.2% (w/v) polygalacturonic acid (sodium salt) for endo-PG. Reduction in viscosity was determined according to a method of Mill and Tuttobello (1961), utilizing a graduated glass pipette as viscometer, which was modified as described in Heerd et al. (2014). The applied sample concentration was adjusted to 1 U/mL of exo-PG activity and the incubation time was reduced to 5 min at 40°C. One unit of endo-pectinase activity (either PG or PMG) was defined according to Patil and Dayanand (2006) as the quantity of enzyme, which caused a 50% reduction in viscosity of the reaction mixture per minute, under the applied assay conditions. Endo-pectinolytic activities were expressed as unit per milliliter crude extract (U/mL).

### Plate assay for proteolytic activity measurement

Dual-substrate assay plates were prepared according to the procedure given by Montville (1983), containing 1% (w/v) casein and 1% (w/v) gelatin as substrates. Wells of 5 mm diameter were cut into the solid media and filled with 30 μL crude extract or commercial pectinase solutions. After 24 h incubation at 30°C the diameters of the zones formed were measured. Zone diameters (D) were converted to log_10_ adjusted zone area by the following expression:

$$log_{10}\;\text{adjusted}\;\text{zone}\;\text{area}\;=\;log_{10}\left[{\left(D/2\right)}^{2}\pi \;–\;{\left(5.0/2\right)}^{2}\pi \right]$$

Proteolytic activity was reported in this manner and referred to as zone area (log_10_ mm^2^).

### Protein pattern analysis

The crude extracts were dialyzed against distilled water over night at 4°C, using SnakeSkin® pleated dialysis tubing, 10,000 MWCO (Thermo Scientific, Rockford, USA). Samples were concentrated to 1.5 mg/mL total protein concentration, using a freeze-dryer.

#### One-dimensional electrophoresis

SDS-PAGE was performed according to the method of Laemmli (1970) as described previously (Heerd et al. 2012). Protein bands were visualized, using colloidal Coomassie (G-250) staining (Neuhoff et al. 1988).

#### Native polyacrylamide gel electrophoresis/zymogram

Native PAGE was performed by excluding SDS and DTT from the electrophoresis protocol described above. The “sandwich” method was used to detect the activity of pectinases acting on polygalacturonic acid sodium salt as substrate (Manchenko 1994). Briefly, proteins were separated on a native PAGE and subsequently the gel was first incubated for 20 min in 0.1 M citrate phosphate buffer (pH 5.0) and afterwards contacted with an (solid) agar substrate containing 0.25% (w/v) polygalacturonic acid sodium salt for 50 min and for 90 min at 30°C (in 80% humidity chamber). The agar plate was then treated with 1% (w/v) cetyltrimethylammonium bromide which precipitated the substrate and revealed pectinase activity as translucent bands on an opaque background.

### Statistical analysis

The software MODDE 9.0 (Umetrics AB, Umeå, Sweden) was used to build and analyze the experimental designs. The mathematic-statistical treatment of the obtained experimental data through the fit of a polynomial function was followed by the evaluation of the model’s fitness, e.g. the two companion statistics, goodness of fit (R^2^) and goodness of prediction (Q^2^) were applied. Furthermore, analysis of variance (ANOVA) was used to evaluate the results for enzymatic activity shown for experimental designs (level of 95% of confidence *p* < 0.05) and evaluation of the model residuals was done using a normal probability plot for detecting deviation experiments. The PG production was analyzed by using a second-order polynomial regression equation for explaining the behavior of the system in optimization experiments.

$$Y=\beta_{0}+\sum_{i=1}^{k}\beta_{i}X_{i}+\sum_{i=1}^{k}\beta_{ii}X_{i}^{2}+\sum_{i}\sum_{j}\beta_{ij}X_{i}X_{j}+\epsilon$$

where Y is the predicted response, k is the number of factor variables, β_0_ the model constant, β_i_ the linear coefficients, X_i_ the independent factor variables, β_ii_ the quadratic coefficients, β_ij_ the interaction coefficients and ϵ is the error factor. The graphical presentation of the polynomial equation in the form of contour plots was used to describe the individual and cumulative effects of the factors on PG production and was obtained via the MODDE 9.0 software as well.

## Results and discussion

### PG production by *A. sojae* ATCC 20235

Results of the first screening investigation are presented in Table 1, exploring the effect of several inducer substrates, inoculum size, temperature, cultivation time and HCl concentration on PG production with a complemented D-optimal design (*R*^*2*^/*Q*^*2*^ 0.88/0.71). The exo-PG activities varied significantly depending on the applied cultivation conditions. Trials performed at high level of HCl concentration resulted in no activity due to the repression of fungal growth, with the exception of experiments utilizing apple pomace as inducer substrate. These trials without fungal growth have been excluded for analysis. The ANOVA results indicated that the most important factors affecting exo-PG activity were the inducer substrates sugar beet pulp and apple pomace as well as the factor HCl concentration. The high moisture content in apple pomace (73%) diluted the total HCl concentration in the medium. In general, acidic pH treatment of media is advantageous in SSF processes applying fungi, due to their tolerance towards low pH-values, which is minimizing the risk of contamination during cultivation. Moreover, many fungi secrete PG in acidic media and this is also the pH range where majority of their PGs show optimum catalytic activity (Niture 2008).

Maximal PG activity of 407.5 ± 13.9 U/g was obtained at the center points of the experimental setup using sugar beet pulp as inducer substrate, which was 1.4 times superior to activities obtained with molassed sugar beet pulp. SSF processes are known to overcome the effect of catabolite repression (Nandakumar et al. 1999, Hölker et al. 2004, Viniegra-González and Favela-Torres 2006). However, the presence of an additional carbon source, like in the case of molasses, might have triggered other metabolic processes than stimulating exo-PG production.

Additionally, to demonstrate the importance of an inducer substrate for pectinase production, *A. sojae* ATCC 20235 was cultivated under conditions of the center point experiments on pure wheat bran and the obtained enzyme activity was about five times lower in comparison to the highest activity obtained with sugar beet pulp as inducer substrate (data not shown). This result strongly favored utilization of pectin-rich inducer substrates, such as sugar beet pulp, in SSF process for pectinolytic enzyme production.

The factor inoculum size had no significant effect on PG production. Based on this fact, in the optimization part of this study the inoculum size was fixed at 2 × 10^7^ total spores, which is close to the level of the center point experiments where maximum PG activity was obtained.

The significance of the factor temperature is known due to its great influence on microbial metabolic activity. Fungi can grow over a wide range of temperatures (20 – 55°C), but optimal temperature for growth can differ from that of product formation (Bhargav et al. 2008). The first screening step provided a survey of temperature ranges and highest PG activity was obtained at the center points incubating at 28°C. Further investigation of this factor was done during the optimization of process parameters.

Due to the exclusion of several experiments resulting in no growth and consequently in no PG production, this first screening investigation provided only an indication for identification of significant factors and their ranges. Therefore, another screening investigation was performed focusing on the most important factors affecting exo-PG activity, HCl concentration and the inducer substrates apple pomace and sugar beet pulp (Table 2). Moreover, the effect of the inducer substrate concentration on PG production was explored, and also the factor incubation time was further investigated using full factorial design (*R*^*2*^/*Q*^*2*^ 0.94/0.73). Even though level of HCl concentration was reduced in the second screening investigation, the applied high level of 0.22 M HCl in combination with high sugar beet pulp concentration inhibited also fungal growth totally (Table 2, trial 8 and 16). These trials have been excluded for analysis. The ANOVA results indicated that both inducer substrates as well as their concentration and, as previously determined, the concentration of HCl significantly influenced exo-PG activity in the crude extracts. Furthermore, the interactions of the inducer concentration/time and HCl concentration/time had a significant effect on enzyme activity, too. Highest PG activity of 374.5 U/g was obtained applying 10% sugar beet pulp as inducer substrate in the medium wetted with 0.22 M HCl after 5.5 days incubation. Utilization of sugar beet pulp as inducer substrate resulted in higher PG activity in comparison to apple pomace, with the exception of those experiments where fungal growth was inhibited. Due to the fact, that the moisture content of these two inducer substrate differed strongly, this affected also the total amount of solid substrate in the SSF process as well as other significant process parameters. The moisture level in SSF systems is very important (Raimbault 1998). High moisture levels decrease the substrate porosity and hence, reduce the oxygen transfer. While low moisture contents may limit the bioavailability of nutrients and increase the accumulation of heat.

Therefore, experiments were conducted comparing PG production with both inducer substrates at similar moisture levels, which was achieved by freeze-drying apple pomace or increasing the moisture content in sugar beet pulp (data not shown). Higher PG activity was obtained utilizing sugar beet pulp, which was chosen as inducer substrate for further optimization investigations. Moreover, longer incubation time indicated further increase of PG activity, which was considered for optimization of this parameter. In addition, further HCl concentrations were tested in the range from 0.175 M to 0.2 M, which resulted in a positive effect on PG production applying HCl concentrations of about 0.2 M (data not shown). The combination of a cultivation medium including predominantly wheat bran and applying the moisture level with 0.2 M HCl concentration was already demonstrated for enhanced microbial enzyme production by filamentous fungus in SSF (Fernandez-Lahore et al. 1997). Therefore, the HCl concentration was fixed at 0.2 M for further optimization experiments.

Summarizing the screening investigations identified sugar beet pulp as potential inducer substrate for PG production in combination with wheat bran and adjusting the moisture level with 0.2 M HCl. Inoculum size in terms of spore concentration had no significant effect on exo-PG activity and was fixed at 2 × 10^7^ total spores.

#### Optimization of PG production by A. sojae ATCC 20235

The first optimization step focused on identification of optimal medium composition investigating the inducer substrate concentration and the moisture level in the medium using a complemented D-optimal design (Table 3). Adjusting the moisture level with 0.2 M HCl influenced water activity and acidity of the fermentation system by varying the factor moisture level. In general, a variable moisture level (as an independent parameter) may seem disadvantageous due to its effect in changing the total reaction volume. A change in the total reaction volume further affects both substrate volume in solid-state cultivation and enzyme concentration obtained after enzyme leaching. The optimal setting of the moisture content factor for cultivation of microorganisms in solid-state cultivation processes is highly dependent upon water-binding properties of the substrate, and the formation of products, such as enzymes, is markedly affected by the moisture content in SSF systems (Prior et al. 1992). Therefore, this factor had to be optimized for PG production by *A. sojae* in the present SSF system.

The original experimental set-up (trial 1 – 11) of the response surface modeling included an inducer concentration range from 10 – 35% and a moisture level range from 85 – 135%. Highest enzyme activity was achieved at high level of moisture and high inducer substrate concentration. Thus, further increase of moisture level and inducer substrate concentration seemed to be promising for higher PG production. The design was complemented by trial 12 – 18, including higher factor ranges up to 65% inducer concentration and 165% moisture level, which created the D-optimal design for response surface modeling.

Trial 16 was detected as outlier and was excluded from optimization analysis. Evaluation of the experimental data identified a sound model quality (*R*^*2*^/*Q*^*2*^ 0.972/0.942). The value of adjusted R^2^ (0.962) was also very high, indicating high significance of the model. Moreover, the lack of fit (LoF) test (*p* = 0.087) pointed in the direction of a valid model. According to the ANOVA results both factors, inducer concentration (X_S_) and moisture level (X_M_), were identified as significant factors, as well as their interaction (X_S_X_M_) and the quadratic term of the variable inducer concentration (X_S_^2^). The response variable may be approximated by the following model equation that expressed exo-PG activity in terms of coded factors:

$$\begin{array}{c} exo‒PG\;\text{activity}\;=\;427.04\;–\;174.56X_{S}\;+\;146.44X_{M}\\ \;–\;90.35X_{S}X_{M}–\;279.46{X_{S}}^{2} \end{array}$$

**Figure 1:**
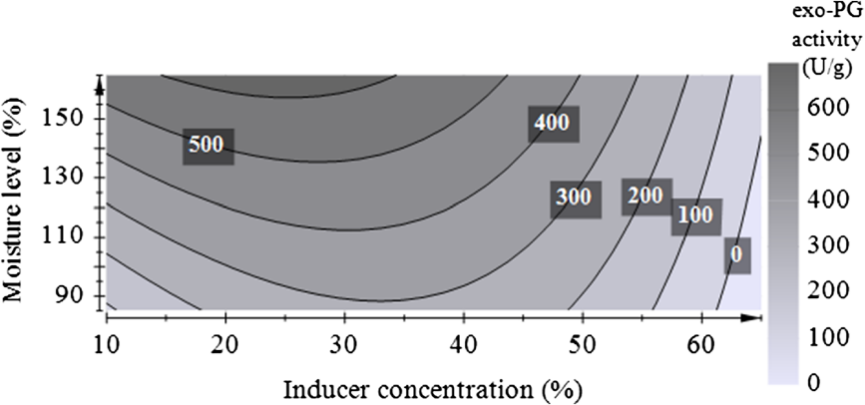
**Response surface plot for PG activity as a function of moisture level and inducer concentration obtained by** ***A. sojae*** **ATCC 20235 (medium optimization with D-optimal design).**

The contour plot obtained from the second-order model is presented in Figure 1.

High PG activities were obtained at trial 4 and 18 using 35% sugar beet pulp as inducer substrate. Prediction for high PG activity values was obtained for inducer concentrations of 15 to 34% and moisture levels of 157 to 165% applied by 0.2 M HCl (Figure 1). Highest PG activity of 613 U/g was predicted at an inducer concentration of 25% and a moisture level of 160%. Therefore, an experimental trial including the predicted optimal conditions for PG production, as well as a slightly higher inducer concentration of 30% closer to the highest results of the optimization experiments were tested. The media were wetted at 160% with 0.2 M HCl and cultivation was performed at 28°C for 5 days. Under the predicted optimal conditions 634.0 ± 44.6 U/g PG activity was obtained which is very close to the predicted result. This represented a very good correlation between the experimental data and the predicted value of the PG activity indicating a good fit of the model. Nevertheless, using 30% of inducer substrate in the medium resulted in a slightly higher PG activity of 648 ± 22.3 U/g. The first optimization step provided an optimized medium composition of wheat bran and sugar beet pulp in the ratio 70:30, wetted at 160% with 0.2 M HCl. The combination of wheat bran and sugar beet pulp in optimized ratio is essential for a productive SSF process by *A. sojae* ATCC 20235. Extracting water soluble proteins and carbohydrates from media blanks containing pure wheat bran or the optimized ratio of wheat bran and sugar beet pulp, yielded higher soluble protein content and higher total soluble carbohydrate concentration in the extract of the mixture with sugar beet pulp (data not shown). This indicated that besides stimulation of pectinolytic enzyme production, the presence of sugar beet pulp as inducer substrate increased also the amount of soluble proteins and carbohydrates available for the fungal metabolism.

The second optimization investigation focused on the process parameters incubation temperature and time. The screening results demonstrated further increase of PG activity at longer incubation times. The original experimental set-up included a time range from 5 to 7 days and a temperature range from 26 to 34°C (Table 4, trial 1 – 11). Due to the tendency of further increase of PG activity at longer cultivation times the experimental set-up was complemented by 3 additional runs with 8 days of incubation time (Table 4, trial 12 – 14). Evaluation of the experimental data upon removal of insignificant model terms identified a sound model quality (*R*^*2*^/*Q*^*2*^ 0.948/0.869). Also the LoF test (*p* = 0.73) strongly pointed in the direction of a valid model. According to the ANOVA results both variables, time (X_t_) and temperature (X_T_), were identified as significant factors, as well as their interaction (X_t_X_T_) and the quadratic term of the variable temperature (X_T_^2^). The response variable may be approximated by the following model equation that expressed exo-PG activity in terms of coded factors:

$$\begin{array}{c} exo‒PG\;\text{activity}\;=\;700.17\;+\;126.51X_{t}–\;65.80X_{T}\\ \;+\;44.40X_{t}X_{T}–\;264.22{X_{T}}^{2} \end{array}$$

**Figure 2:**
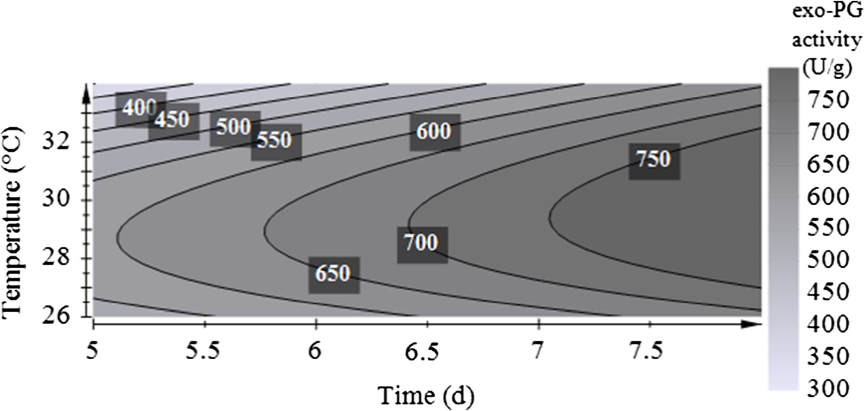
**Response surface plot for PG activity as a function of temperature and time obtained by** ***A. sojae*** **ATCC 20235 (process optimization with D-optimal design).**

**Figure 3:**
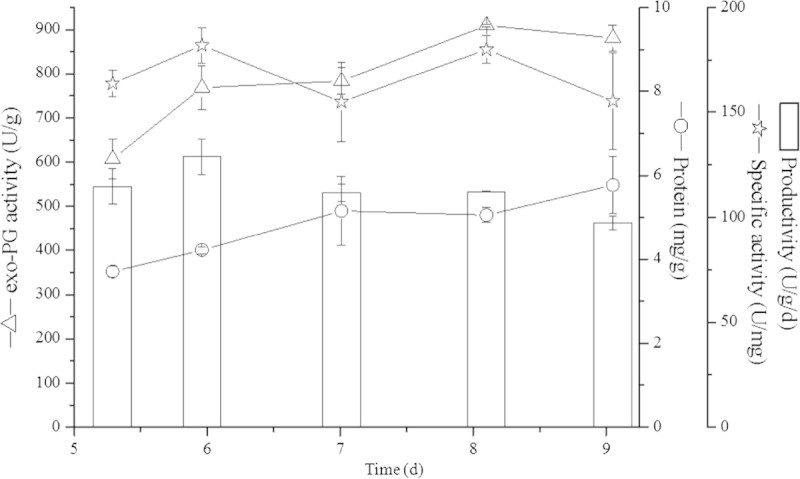
**Solid-state fermentation profile of** ***A. sojae*** **ATCC 20235 utilizing optimal conditions for exo-PG production.**

Maximal exo-PG activity of 847.3 U/g was obtained after 8 days at 30°C (Table 4, trial 13). The contour plot seen in Figure 2 was constructed from the model for exo-PG activity and demonstrates that high PG activity at different cultivation times is given at the temperature scale around 30°C. However, the results also strongly indicated further increase of PG activity at longer incubation times. Therefore, further investigation on the factor cultivation time was done to determine the optimal conditions for PG production by *A. sojae* ATCC 20235 in SSF. Incubation temperature was fixed at 30°C and cultivation was performed over a period of nine days harvesting daily samples from day 5 till day 9 (Figure 3). According to the results obtained between five to nine days, the maximum of PG activity was achieved at the 8th day of cultivation with 909.5 ± 2.7 U/g. Comparing this value with PG activity obtained during the optimization investigation at trial 13 with 847.3 U/g, the variation is below 7% which indicated also a good reproducibility. At the peak of enzyme production also a high specific activity of 180.0 ± 6.8 U/mg protein was obtained. The maximal productivity of 128.9 U/g/d was achieved after six days, it slightly decreased to 112.3 ± 0.3 U/g/d at the 8th day of SSF. Only the amount of total protein in the crude extract was further increasing over the time. With regard to previous work (Heerd et al. 2012), where the potential of this strain as PG producer in SSF was presented utilizing a mixture of wheat bran and dried orange peel wetted at 120% with 0.2 M HCl, the maximum in exo-PG activity was 10.9 times increased by this optimization, measuring both extracts under the same enzyme assay conditions described in the present study. Productivity determined at the peak of enzyme activity was 6.7 fold increased after optimization.

### PG production by *A. sojae* CBS 100928 in SSF

Prior to this statistical optimization study a screening on different inducer substrates was performed and maximal exo-PG activity was obtained with sugar beet pulp (data not shown). Thus, the optimized medium for PG production by *A. sojae* ATCC 20235, which contained wheat bran and sugar beet pulp in the ratio 70:30, wetted with 0.2 M HCl, provided the basis medium for optimizing enzyme production by *A. sojae* CBS 100928.

Table 5 presents the results obtained for exo-PG activity in the full factorial design (*R*^*2*^/*Q*^*2*^ 0.97/0.84) with three variables studied: moisture level, temperature and cultivation time. The screening data indicated that high values of enzyme activity were obtained at incubation times of six to eight days in combination with higher moisture levels and incubation temperature of 30°C. Maximum exo-PG activity of 108.1 ± 5.9 U/g was achieved at the center point trials at 30°C. Therefore, the temperature was fixed at 30°C and conditions of moisture level and cultivation time were further optimized for enhanced enzyme production.

#### Optimization of PG production by A. sojae CBS 100928

Table 6 shows the optimization results obtained for exo-PG activity in CCF design with the two studied variables moisture level and time. Evaluation of the experimental data upon removal of insignificant model terms identified a sound model quality (*R*^*2*^/*Q*^*2*^ 0.97/0.815). Also the LoF test (*p* = 0.485) pointed in the direction of a valid model.

According to the ANOVA results both factors, moisture level (X_M_) and time (X_t_), were identified as significant factors, as well as their interaction (X_M_X_t_) and the quadratic term of the variable moisture level (X_M_^2^). The response variable may be approximated by the following model equation that expressed exo-PG activity in terms of coded factors:

$$\begin{array}{c} exo‒PG\;\text{activity}\;=\;102.78\;–\;11.52X_{M}\;+\;13.45X_{t}\\ \;+\;9.50X_{M}X_{t}–\;21.73{X_{M}}^{2} \end{array}$$

**Figure 4:**
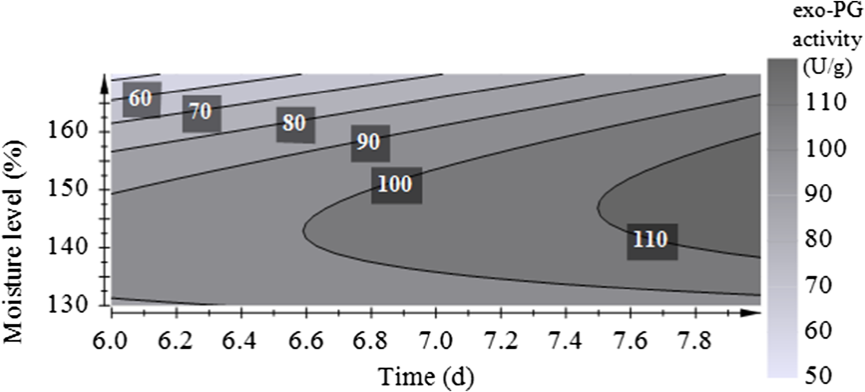
**Response surface plot for PG activity as a function of moisture level and time obtained by** ***A. sojae*** **CBS 100928 (optimization with CCF design).**

The contour plot obtained from the second-order model is presented in Figure 4, where high enzyme activity was predicted at moisture levels of 138 to 160% and incubation times from 7.5 to 8 days. Optimal conditions for PG production in SSF by *A. sojae* CBS 100928 were similar to the optimized conditions of *A. sojae* ATCC 20235. Maximal PG activity in optimization trials was obtained in trail 8, wetting the substrate at 150% with 0.2 M HCl and incubating for 8 days at 30°C.

Validation experiments were conducted at the predicted optimal point (conditions of trail 8) and experiments applying a moisture level of 160%, which represented the optimized conditions for the other strain. The predicted enzyme activity under optimal conditions was 116.3 U/g and achieved was an exo-PG activity of 131.9 ± 6.9 U/g, which indicated a good compatibility of the model with the experimental results. PG production at a moisture level of 160% was slightly lower with 118.1 ± 1.2 U/g. Hence, optimized conditions for exo-PG production by *A. sojae* CBS 100928 in SSF utilizing a mixture of wheat bran and sugar beet pulp in the ratio 70:30 as substrate were determined at a moisture level of 150% applied by 0.2 M HCl after 8 days incubation at 30°C.

### Comparison under optimized conditions

**Figure 5:**
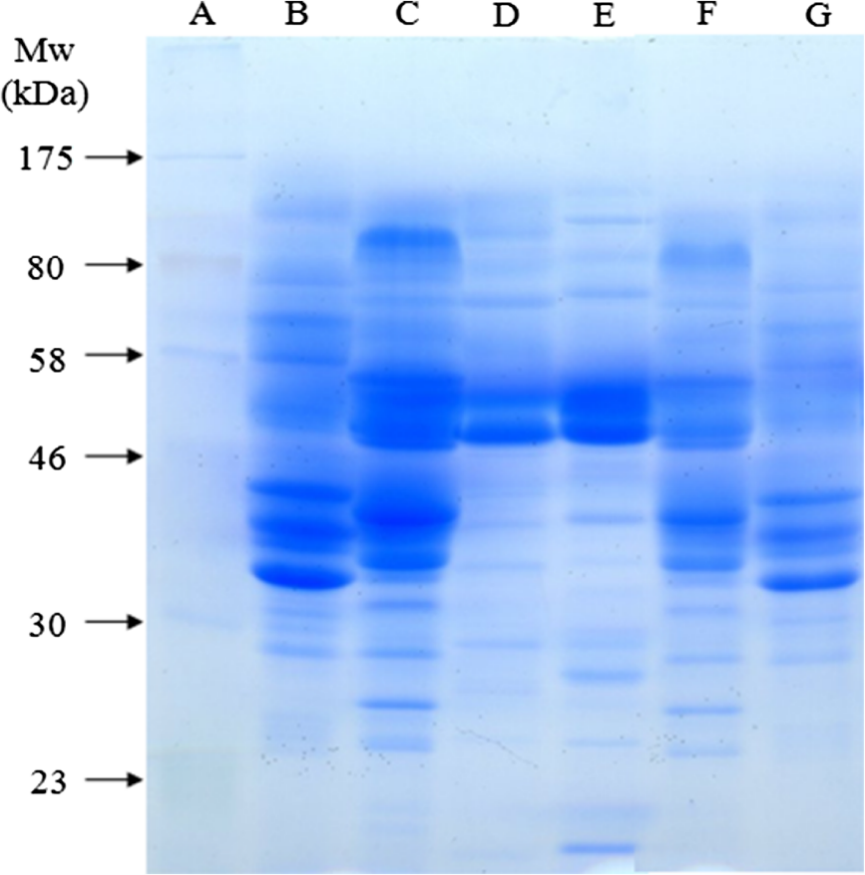
**Analysis of crud extracts obtained under optimized conditions for pectinase production by SDS-PAGE: lane A: ColorPlus prestained protein marker (New England BioLabs); lane B: commercial pectinase (SIGMA); lane C: Fructozym P (Erbslöh); lane D:** ***A. sojae*** **CBS 100928; lane E:** ***A. sojae*** **ATCC 20235; lane F: Fructozym P (1:2 dilution); lane G: commercial pectinase (1:2 dilution).**

Comparing enzyme activities obtained under optimized conditions, 6.9 times higher pectinase activity was achieved by *A. sojae* ATCC 20235. This result confirmed previous findings of highest pectinase production by *A. sojae* ATCC 20235 in SSF (Heerd et al. 2012). Highest fungal polygalacturonic acid degrading exo-enzyme activity (480 U/g) measured with the arsenomolybdate reagent in SSF extracts was produced on wheat bran by *A. carbonarius* (Jacob 2009). This enzyme yield was 1.9 times increased using *A.* sojae ATCC 20235 in SSF under optimized conditions in this study. Crude extracts of *A. sojae* ATCC 20235 and *A. sojae* CBS 100928 obtained under optimized conditions for pectinolytic enzyme production were analyzed by polyacrylamide gel electrophoresis under denaturing conditions. Results are presented in Figure 5. The profile of both fungal strains presented two remarkable bands between the protein standards of 46 kDa and 58 kDa. Especially noticeable is the increase of these bands in the extract of *A. sojae* CBS 100928 after optimizing the pectinolytic enzyme production in comparison to previous results of Heerd et al. (2012). Comparing these bands to the protein profile of commercial pectinase preparations suggested the presence of a protein band at the same level of the lower thick band in Fructozym P (Erbslöh Geisenheim AG), which is a concentrated pectinolytic enzyme preparation for pectin degradation in fruit mash and fruit juice.

**Figure 6:**
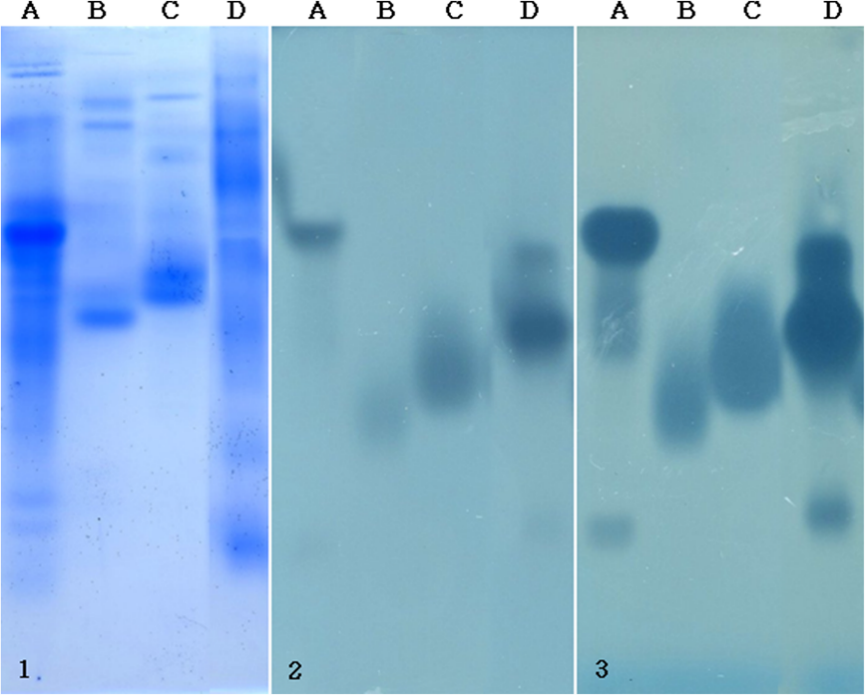
**Native PAGE:** **(1) Electrophoresed gel and (2) substrate containing agar plate after 50 min pre-incubation, (3) substrate containing agar plate after 90 min pre-incubation; Lane A: Fructozym P (Erbslöh); lane B:** ***A. sojae*** **CBS 100928; lane C:** ***A. sojae*** **ATCC 20235; lane D: commercial pectinase (SIGMA).**

The protein content of the extracts was also fractionated by native electrophoresis so as to preserve enzyme activity (Figure 6). It has to be recalled that the active fractions (bands or zones) observed in this case are not directly related to the molecular mass since separation in native gel is related to charge/size. This makes the information gathered in Figure 6 difficult to compare with the profiles observed in Figure 5. Nevertheless, it can be noticed that the extracts of the different strains induced a distinctive pattern in the zymogram. In contrast to previous findings (Heerd et al. 2012), only one active zone was observed in the extract produced under optimized conditions by *A. sojae* CBS 100928. Based on the results of this study SSF process conditions and media design significantly influence protein pattern produced in SSF and hence enzyme production. Moreover, comparing the electrophoresed gel with the substrate-containing agar plate in Figure 6 revealed the presence of protein bands in the extracts of both fungal strains, which did not produce active zones on polygalacturonic acid sodium salt as substrate. This indicated the production of other enzyme types under the optimized conditions. The commercially available enzyme preparations from Sigma-Aldrich and Erbslöh Geisenheim AG were produced by *Aspergillus spp.* in SmF and presented protein patterns with several active zones (Figure 6). Commercial pectinase preparations usually contain mixtures of various pectinolytic enzymes, which are additionally associated with cellulolytic, proteolytic and other species of enzymes apart from the main pectinases (Del Cañizo et al. 1994). Thus, comparing the electrophoresed gel with the substrate-containing agar plate in Figure 6 also revealed the presence of protein bands in the commercial pectinase preparations, which did not produce active zones. The composition of enzyme sets differ significantly between fungal species and this is also observed for the subset of pectinolytic enzymes (Benoit et al. 2012). The difference in enzyme production between SSF and SmF was already discussed in a recent study (Kumar et al. 2011).

### Comparison of enzyme profiles

Investigation of pectinolytic enzyme sets was performed on enzyme extracts obtained from both strains under optimized conditions for PG production applying a moisture level of 160% (Table 7). The difference in exo-PG activity in comparison to the optimization results might be caused by utilization of sugar beet pulp from another harvest batch. This could imply that the beet root was harvested from another producer region and also at another season. These factors would strongly effect the composition of beet roots, e.g. by varying the sugar content, and hence the type of inducer substrate.

**Table 7:** **Comparison of enzyme activities obtained under the same cultivation conditions**

Microorganism	PG activity (U/g)	Specific activity (U/mg)	endo-activity (U/mL) at 1 U/mL exo-PG activity		PMG activity (U/g)	Proteolytic activity (log_10_mm^2^)
	exo	exo-PG	PG	PMG	exo	
*A. sojae* ATCC 20235	632.7 ± 61.7	133.7 ± 10.2	1.08 ± 0.07	0.87 ± 0.08	30.1 ± 2.4	2.50 ± 0.05
*A. sojae* CBS 100928	133.2 ± 12.1	54.6 ± 7.7	1.13 ± 0.01	0.99 ± 0.01	20.7 ± 1.9	2.12 ± 0.02

The observed values of enzyme activities were the mean values of duplicates with standard deviation (mean ± SD).

Exo-PMG activity obtained by cultivation of *A. sojae* ATCC 20235 was higher compared to *A. sojae* CBS 100928. In comparison to results of previous work (Heerd et al. 2012), PMG activity in the extract of *A. sojae* ATCC 20235 slightly decreased by 10%, while the PMG activity in the extract of *A. sojae* CBS 100928 increased by 25% after optimizing SSF conditions. These changes are irreducible in contrast to the change of exo-PG activity, which is confirming that primarily the exo-pectinase activity degrading polygalacturonic acid was increased by this optimization. Furthermore, the obtained extracts of both strains were diluted to 1 U/mL of exo-PG activity to compare the ratio of exo-PG activity and endo-pectinolytic activities. The values given in Table 7 show an almost balanced ratio of exo- to endo-PG activity for both strains with a slightly higher endo-PG activity. The ratio of exo-PG activity to endo-PMG activity is also balanced in the extract of *A. sojae* CBS 100928. The endo-PMG activity in the extract of *A. sojae* ATCC 20235 is slightly lower than the exo-PG activity. The SSF process optimization for enhanced exo-PG production increased also the endo-enzyme activities of both strains in comparison to previous results (Heerd et al. 2012). Comparing endo-PG with endo-PMG activities, higher activity of polygalacturonic acid degrading enzymes was produced in the extracts of both strains.

In addition to pectinolytic activities also the proteolytic activity was determined. Activity of proteases in the extract of *A. sojae* ATCC 20235 was higher than in the extract of *A. sojae* CBS 100928. In comparison to previous results, no significant changes in proteolytic activity were observed (Heerd et al. 2012), which indicated that the process optimization had no influence on proteolytic enzyme production.

Comparing present results with reported PG activities in the literature, previous optimization studies with *A. sojae* ATCC 20235 applying crushed maize wetted with a nutrient solution, which resembled a surface cultivation, yielded 29.1 U/g exo-PG activity (Ustok et al. 2007). PG production by *A. sojae* ATCC 20235 was dramatically increased under solid-state conditions in the present study optimizing the SSF process and utilizing a cost-efficient medium.

Utilization of agro-based products in SmF and using a mutant of this strain, generated by treatment with UV radiation, yielded an enzyme activity of 145.4 U/mL in shaking flask cultures (Buyukkileci et al. 2011). The combination of optimizing the fermentation process utilizing agricultural substrates with microbial strain improvement increased dramatically the enzyme yield in SmF. The results of the present optimization study showed already the potential of PG production by the wild type in SSF utilizing agricultural products. The combination with microbial strain improvement might generate a process of PG production which could be attractive for industrial applications.

## Conclusions

PG production by *A. sojae* was optimized applying crude plant compounds, such as wheat bran and sugar beet pulp, as substrate in SSF. The present study demonstrated a great potential for cost-efficient pectinolytic enzyme production by *Aspergillus sojae* ATCC 20235. Production under optimized conditions in laboratory scale yielded high exo-PG activity and sugar beet pulp was identified as significant pectinase inducer substrate. Utilization of agricultural and agro-industrial by-products developed an attractive sustainable bioprocess for enzyme production.

Optimized pectinolytic enzyme production yield of *A. sojae* ATCC 20235 was 6.9 times higher in comparison to *A. sojae* CBS 100928. High enzyme yield obtained by *A. sojae* ATCC 20235 under optimized conditions will be a promising starting point for scale-up and polygalacturonase purification studies.

## Authors’ original submitted files for images

Below are the links to the authors’ original submitted files for images. Authors’ original file for figure 1 Authors’ original file for figure 2 Authors’ original file for figure 3 Authors’ original file for figure 4 Authors’ original file for figure 5 Authors’ original file for figure 6
