# Supplementary figures and images for: Efficient polygalacturonase production from agricultural and agro-industrial residues by solid-state culture of Aspergillus sojae under optimized conditions
Source: Springerplus. 2014 Dec 16;3:742. doi: 10.1186/2193-1801-3-742 (PMC4320240; doi:10.1186/2193-1801-3-742)

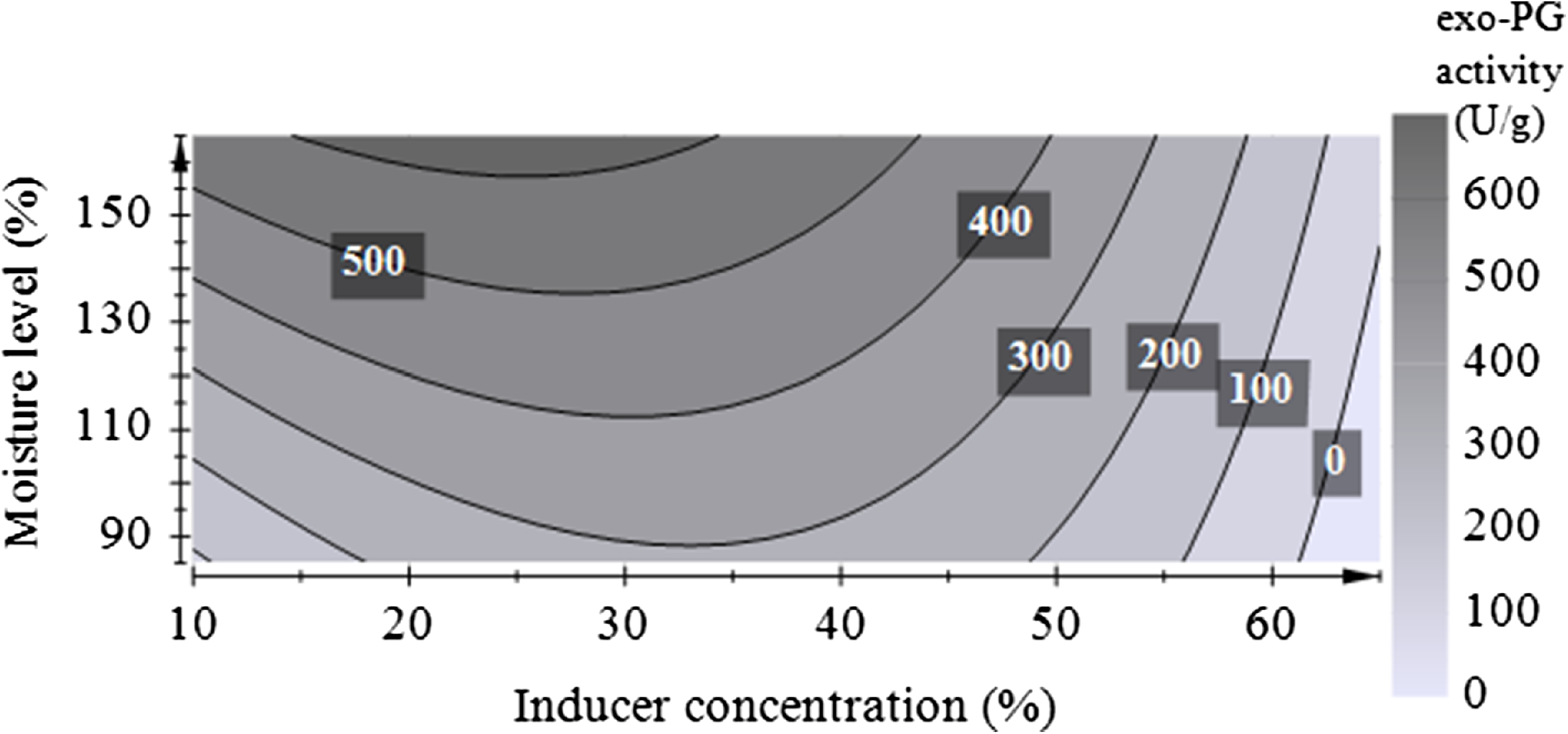

Supplement: Supplementary file 1 — Authors’ original file for figure 1 [file 40064_2014_1501_MOESM1_ESM.tif]

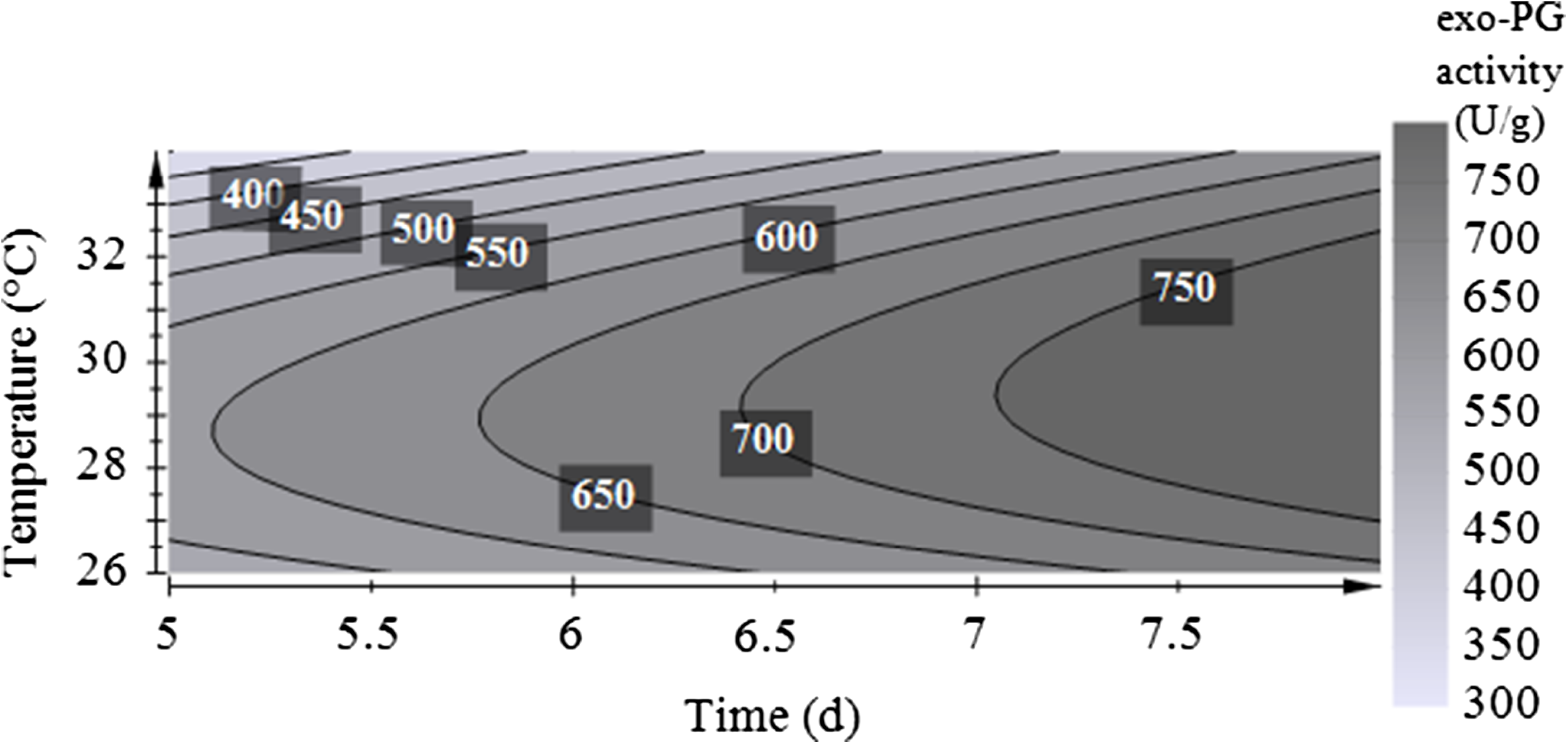

Supplement: Supplementary file 2 — Authors’ original file for figure 2 [file 40064_2014_1501_MOESM2_ESM.tif]

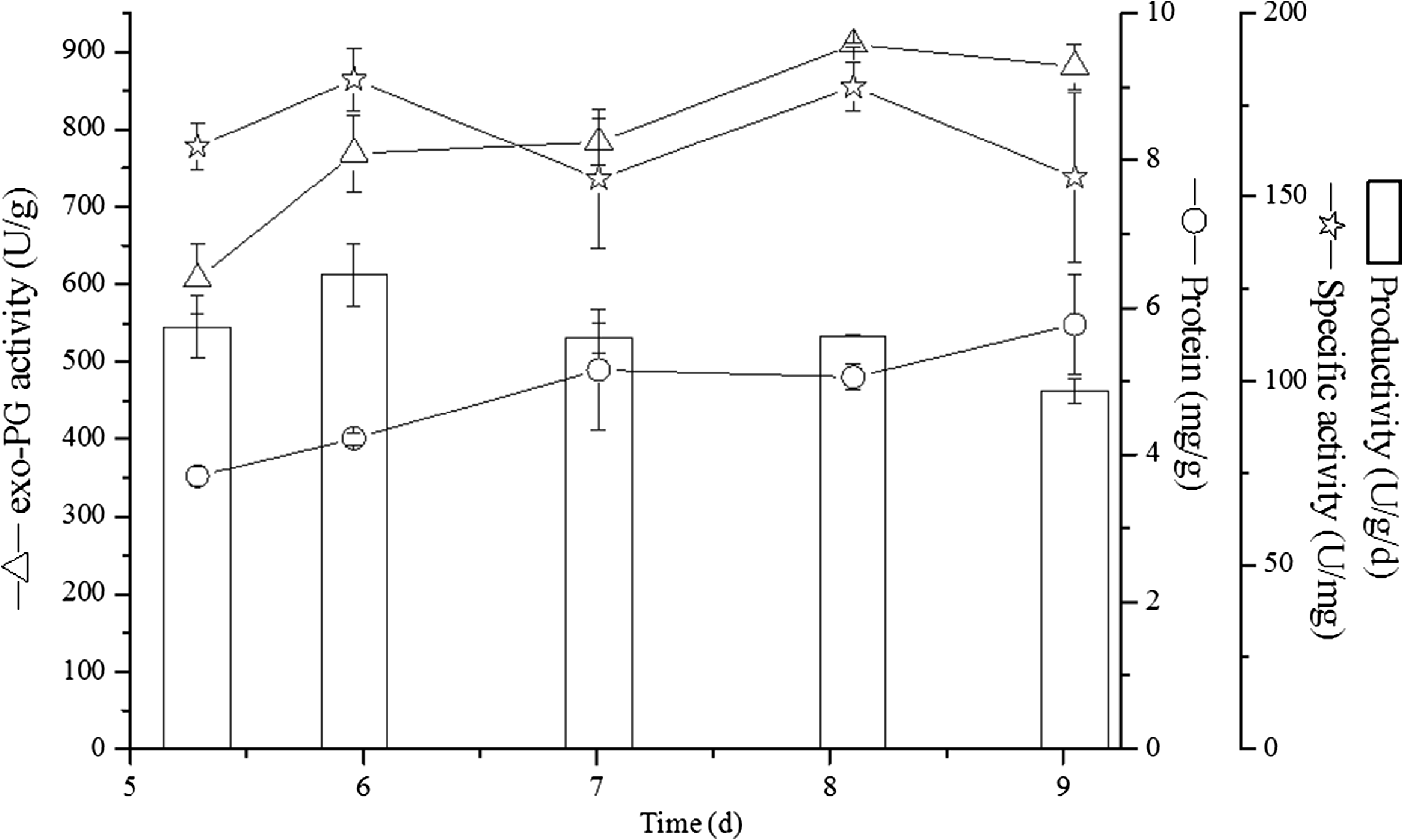

Supplement: Supplementary file 3 — Authors’ original file for figure 3 [file 40064_2014_1501_MOESM3_ESM.tiff]

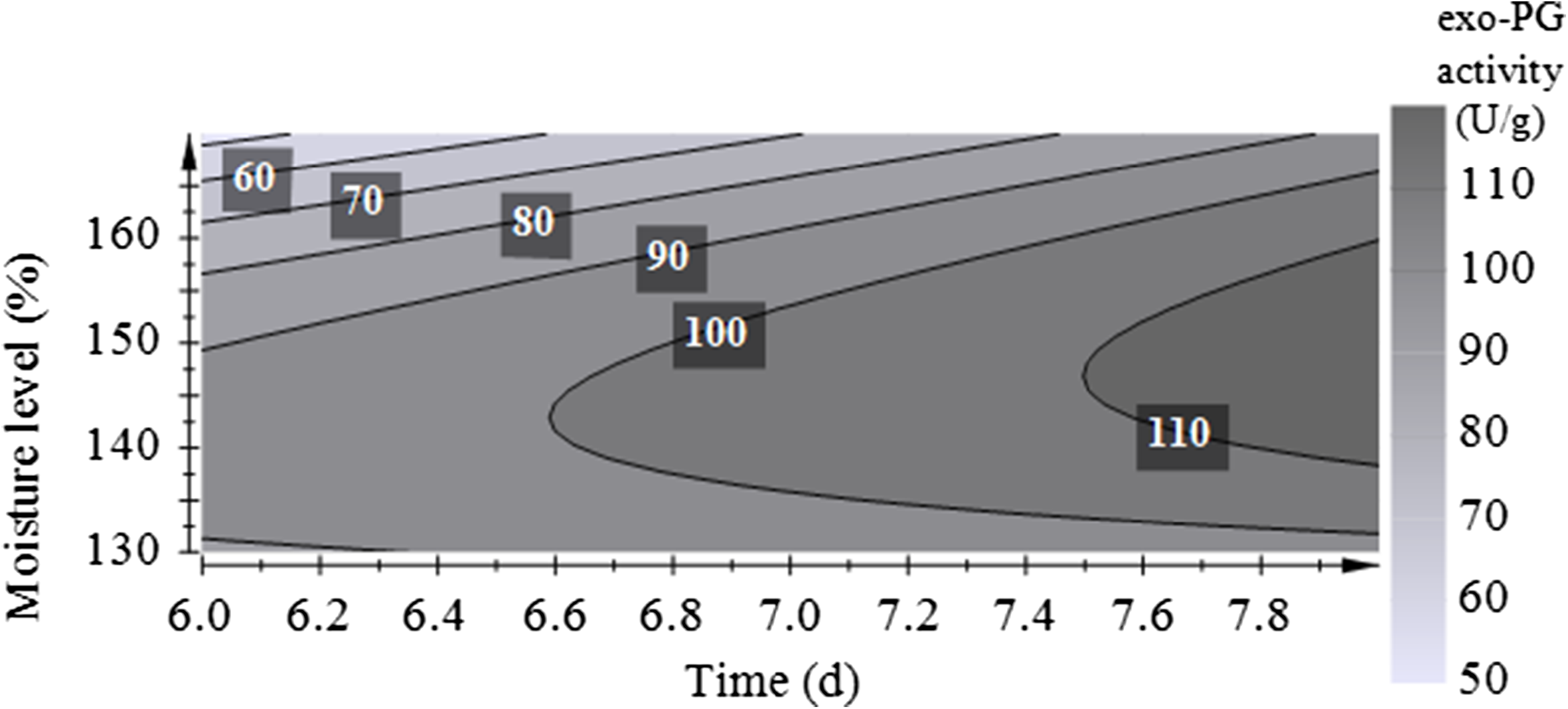

Supplement: Supplementary file 4 — Authors’ original file for figure 4 [file 40064_2014_1501_MOESM4_ESM.tiff]

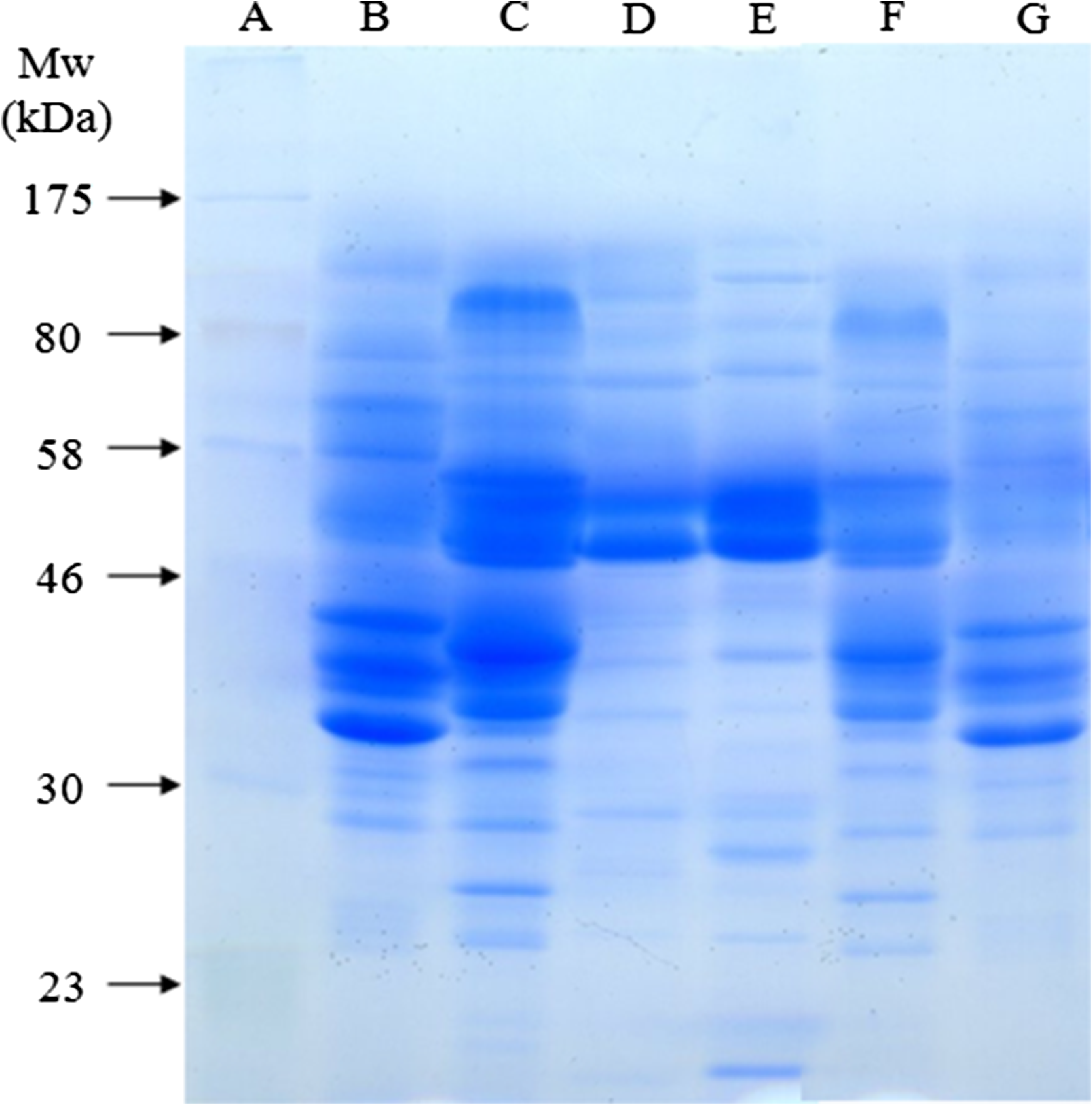

Supplement: Supplementary file 5 — Authors’ original file for figure 5 [file 40064_2014_1501_MOESM5_ESM.tif]

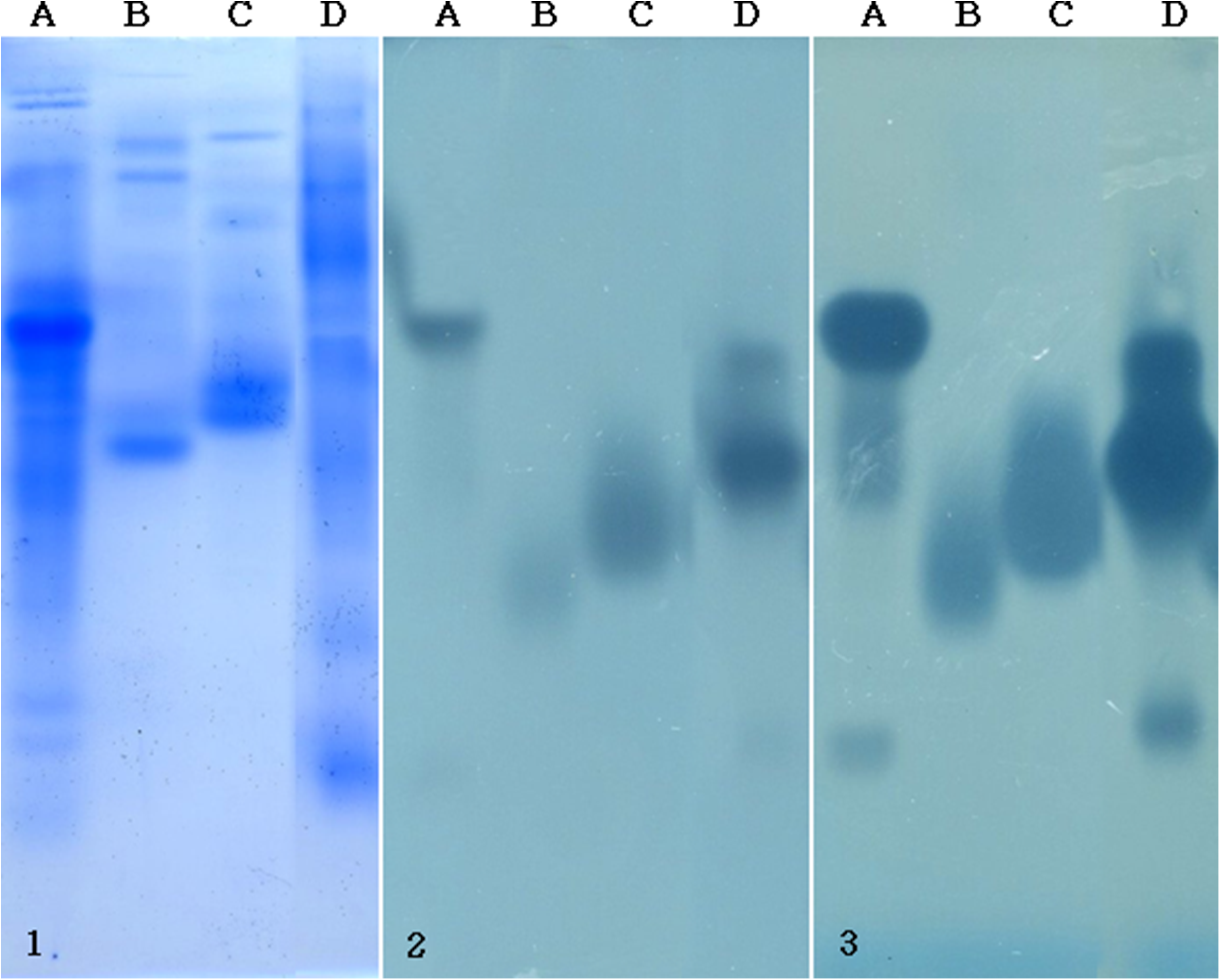

Supplement: Supplementary file 6 — Authors’ original file for figure 6 [file 40064_2014_1501_MOESM6_ESM.tif]
